# Coastal subsidence increases vulnerability to sea level rise over twenty first century in Cartagena, Caribbean Colombia

**DOI:** 10.1038/s41598-021-98428-4

**Published:** 2021-09-23

**Authors:** Juan D. Restrepo-Ángel, Héctor Mora-Páez, Freddy Díaz, Marin Govorcin, Shimon Wdowinski, Leidy Giraldo-Londoño, Marko Tosic, Irene Fernández, Juan F. Paniagua-Arroyave, José F. Duque-Trujillo

**Affiliations:** 1 Department of Earth Sciences, School of Sciences, Universidad EAFIT, AA 3300 Medellín, Colombia; 2 Colombian Geological Survey, Space Geodesy Research Group, Bogotá, Colombia; 3 Faculty of Geodesy, Institute of Geomatics, University of Zagreb, Zagreb, Croatia; 4 Department of Earth and Environment, Institute of Environment, Florida International University, Miami, USA

**Keywords:** Physical oceanography, Natural hazards

## Abstract

Cartagena is subsiding at a higher rate compared to that of global climate-driven sea level rise. We investigate the relative sea level rise (RSLR) and the influence of vertical land movements in Cartagena through the integration of different datasets, including tide gauge records, GPS geodetic subsidence data, and Interferometric Synthetic Aperture Radar (InSAR) observations of vertical motions. Results reveal a long-term rate (> 60 years) of RSLR of 5.98 ± 0.01 mm/yr. The last two decades exhibited an even greater rate of RSLR of 7.02 ± 0.06 mm/yr. GPS subsidence rates range between − 5.71 ± 2.18 and − 2.85 ± 0.84 mm/yr. InSAR data for the 2014–2020 period show cumulative subsidence rates of up to 72.3 mm. We find that geologically induced vertical motions represent 41% of the observed changes in RSLR and that subsidence poses a major threat to Cartagena’s preservation. The geodetic subsidence rates found would imply a further additional RSLR of 83 mm by 2050 and 225 mm by 2100. The Colombian government should plan for the future and serve as an example to similar cities across the Caribbean.

## Introduction

**Figure 1:**
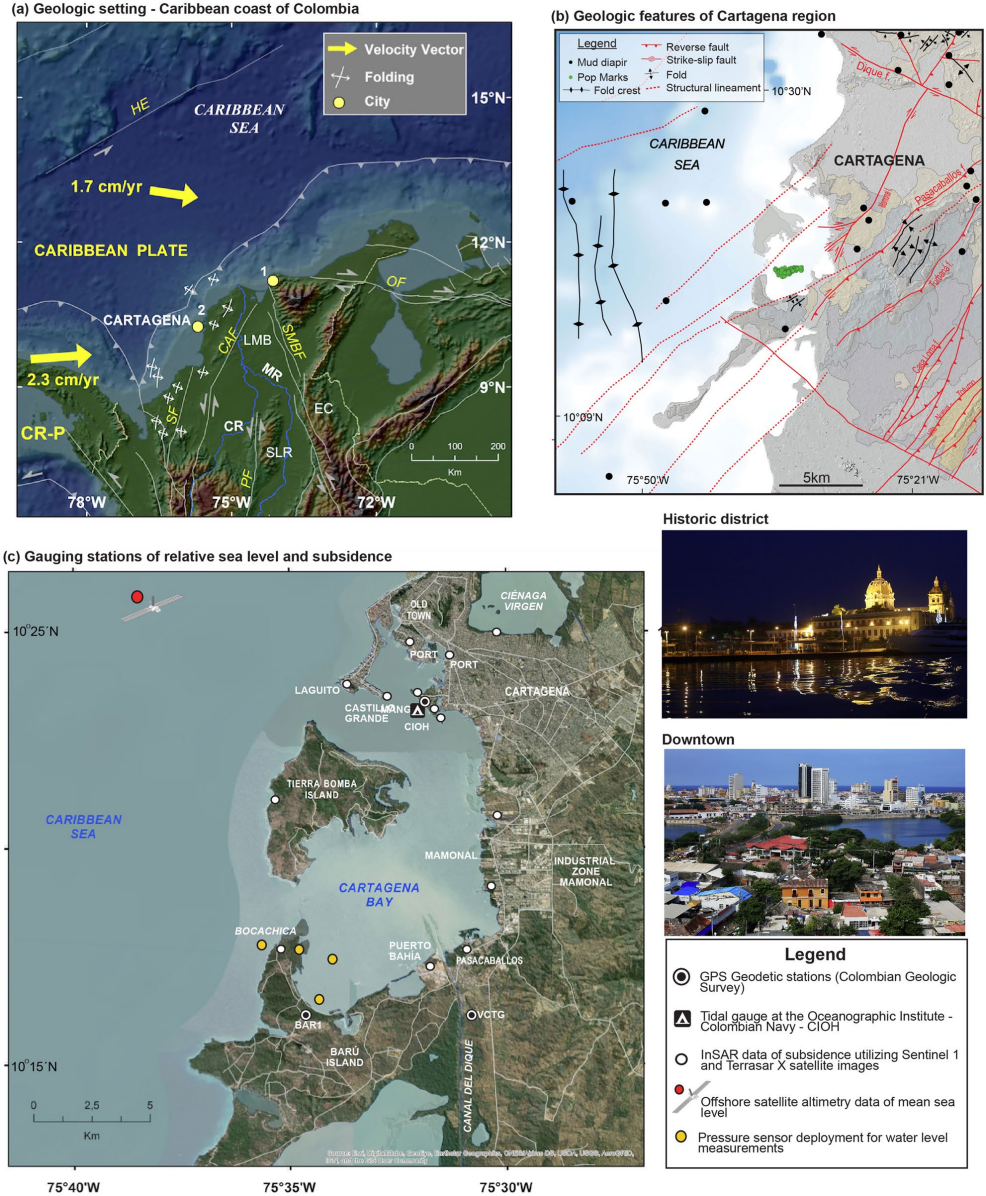
Geologic setting and sampling stations. (**a**) Geologic setting of the Caribbean coast of Colombia showing major tectonic plates and faults, mountain ranges, rivers, and coastal cities (CR-P: Costa Rica Panamá Microplate; Cr: Cocos Ridge; CR: Cauca River; MR: Magdalena River; SLR: San Lucas Range; EC: Eastern Cordillera; SF: San Jeronimo Fault; PF: Palestina Fault; SMBF: Santa Marta Bucaramanga Fault; OF: Oca Fault; Cities: 1. Santa Marta; 2. Cartagena; 3. Panama City). GPS velocities relative to South America plate. (**b**) Main geologic features that produce tectonic-induced vertical motions in Cartagena region, including mud volcanoes and diapirs (see Supplementary Fig. S2), associated pop marks, and tectonic faults. (**c**) Map of Cartagena city and its bay, showing the main gauging stations of relative seal level and land subsidence used in this study. (**a**–**c**) maps generated by ArcMap from ESRI, https://desktop.arcgis.com/es/arcmap/ (*Photo credits of Cartagena – Juan D. Restrepo, IDRC-BASIC Project).*

The coastal city of Cartagena, Colombia (Fig. 1), has approximately one million inhabitants, a large number of prominent ports and shipping operations, and the country's largest coastal industrial sector. The historic city, its nearby beaches and marine protected area combine to represent Colombia's principal touristic destination^1^. However, the city’s recent success in marketing itself may be undermining the sustainability of its own tourism sector. In 2019, more than 500,000 foreign tourists visited, triple the number of 2012, while domestic visitors still outnumber them. A study commissioned by UNESCO warns that “the intensive use of tourism” threatens the city’s preservation^2^. Here, we show that subsidence-induced sea level rise poses a major threat to Cartagena’s preservation, tourism, infrastructure and vulnerable coastal communities.

Many of the world’s coastal cities are sinking faster than the eustatic rise of sea level^3^. Coastal land sinking, or subsidence, occurs naturally in the absence of anthropogenic processes^3–6^ through sediment compaction and vertical tectonic movement of the earth’s crust. In addition to the reduction of sediment load into the coastal zone, tectonic processes result in changes to surface elevation through uplift or subsidence of the entire sediment column^5^.

Local subsidence, which can exceed absolute sea level rise (ASLR) by one order of magnitude, is the main driver of RSLR in many coastal cities^6–11^, islands^12,13^, and deltas^5,8,14,15^ around the world. Quantitative estimates of subsidence and understanding its controlling mechanisms are of primary importance to assess the impacts of contemporary and future rates of RSLR on global low lying coastal cities and deltas^8^. In addition, developing mitigation plans for subsidence and associated rising sea levels requires a combination of measurement and monitoring strategies. The spatial and temporal changes in land elevation associated with vertical motions must be estimated accurately. A further challenge is to separate out the processes that contribute to relative sea level change across coastal cities^3^.

While global climate change and associated sea level rise have received much scientific attention around the world’s coastal regions^16–18^, naturally induced subsidence and its effect on RSLR in complex tectonic coastal regions have gotten comparatively little consideration. In the Caribbean, there are no published rates of coastal subsidence, nor analyses of the contribution of vertical land motion to RSLR. Most studies have analyzed sea level trends, their variability and extremes^19–23^. Overall, the Caribbean mean sea level rose at an average rate of 1.8 mm/yr from 1950 to 2009^23^ and 1.7 ± 1.4 mm/yr during the 1993–2010 period^20^. After glacial isostatic correction, the basin average trend of RSLR is 2.5 ± 1.3 mm/yr^20^.

Relative sea level trends in Cartagena have been addressed in the above-mentioned works and other studies^21,24,25^. A regional sea level trend assessment in the Caribbean Sea, covering 19 tide gauge stations from Cuba down to Panama, revealed that Cartagena has the highest trend (5.3 ± 0.3 mm/yr), almost three times greater than the RSLR observed at the nearest station in Cristobal, Panama (1.9 ± 0.3 mm/yr). This study noted that the RSLR trend in Cartagena could be affected by local vertical land movements^20^. Previous studies also found evidences of subsidence in the city of Cartagena based on GPS data obtained in stations located in the same area of the tide gauge^25–27^. The Nevada Geodetic Laboratory provided a more recent estimation for the vertical component at one of these stations, CART (− 2.11 ± 0.74 mm/yr), expressed in IGS14, with 20 years of observation, however, the time series show various periods of missing data (http://geodesy.unr.edu/NGLStationPages/stations/CART.sta).

Our understanding of the patterns in spatial and temporal variability in subsidence rates in Cartagena has been limited due to the lack of reliable data and an integrated strategy to combine remote sensing with a terrestrial network of site-specific measurement stations, similar to the strategies implemented in other cities with large subsidence rates (e.g., New Orleans)^3^. This limitation has made it difficult to quantify the role that subsidence has played on RSLR as well as its contribution to the loss of 342 ha of mangroves in Cartagena Bay^25^. Also, previous analyses of sea level trends in Cartagena have indicated that the large component of land movement in the city is probably due to sediment compaction in the coastal spit of Castillogrande, an area which has been massively urbanized with tall buildings since the 1970’s^20,28^. However, further studies are needed to resolve whether this land sinking is restricted to this region or if the issue extends further to the protected heritage site of the city and coastal areas, including the ports and industries^20^.

On a regional scale, sea level trends in the Caribbean appear to be dominated by sub-basin and local processes^20^. Therefore, global and even regional values of sea level change are not adequate to be applied in coastal planning and protection guidelines, nor in the estimation of coastal vulnerability^20^. Most mitigation plans for rising sea levels in Cartagena are developed using global trends of mean sea level^29^. In fact, environmental authorities, policy makers, and stakeholders blame climate change as the main cause of rising sea levels and associated flood hazards. In contrast, we demonstrate that under the future scenarios of sea level rise of 25–30 cm by 2050^29–32^, Cartagena is many times more vulnerable to local-scale RSLR than it is to rising global sea level due to climate change.

Here, we investigate the trends in RSLR and identify the contribution of vertical land motion on RSLR in Cartagena. We use the most up-to-date data available in the region from offshore satellite radar altimetry, tide gauge logs, GPS, interferometric Synthetic Aperture Radar (InSAR), pressure sensors as well as sea level projections for 2050 and 2100 (Fig. 1c). Future city planning, including the conservation of cultural heritage, flood mitigation of coastal communities, and infrastructure development, must implement consistent subsidence measurements and modeling across the city, in order to link science with the socioeconomic implications of RSLR.

## Results

### Geologic setting of Cartagena

The Caribbean coastal margin of Colombia is a geologically complex region where tectonic movements have defined a physiographic setting of contrasting landscape units, including extensive low-relief deltaic plains and medium- to high-relief mountainous areas^33–35^. The entire coastline, including the region of Cartagena, is located in an active tectonic zone where the Caribbean and South American plates converge (Fig. 1a). The geomorphology has been deeply influenced by numerous offshore and onshore active diapirs and mud volcanoes (see Supplementary Fig. S2) evidenced by weakened rock zones and domes (Fig. 1b), several of them with historical records of violent mud eruptions often triggered by seismic events^36^. All these mud volcanic formations and associated eruptions have been correlated with vertical land motions along the coast due to fluid release, sediment compaction and associated subsidence^37–39^. Also, Cartagena is largely influenced by many reverse and strike-slip faults that produce both compressional and transpressional tectonism (Fig. 1b). There is a strong consensus in the Colombian scientific community that the Caribbean coast, including the zone of Cartagena, is an active tectonic area characterized by unstable coastal lands and subsidence events associated with mud diapir-volcanoes and faults^37–39^. Further geologic descriptions of mud diapirism and recent tectonism in Cartagena are included in the Supplementary Information.

### Relative seal level

Monthly Absolute Sea Level (ASL) was obtained from AVISO (Archiving, Validation and Interpretation of Satellite Oceanographic data^40^, http://www.aviso.altimetry.fr) for the 1993–2015 period. The altimetry data reveal an increasing ASL trend of 3.18 ± 0.29 mm/yr for Cartagena’s offshore area (see Supplementary Fig. S3). The steric effects on eustatic sea level is about 50%.

Tide gauge measurements are the main data source for coastal sea level changes since the mid-nineteenth century. These gauges estimate relative sea level (RSL), which is the sea level relative to the land on which they are located. We obtained monthly RSL records during the 1952–2000 period from the tide gauge in Cartagena from the University of Hawaii Sea Level Center (UHSLC, https://uhslc.soest.hawaii.edu/). Hourly records for the 2001–2019 period were obtained from the Oceanographic and Hydrographic Research Institute (CIOH) in Cartagena.

**Figure 2:**
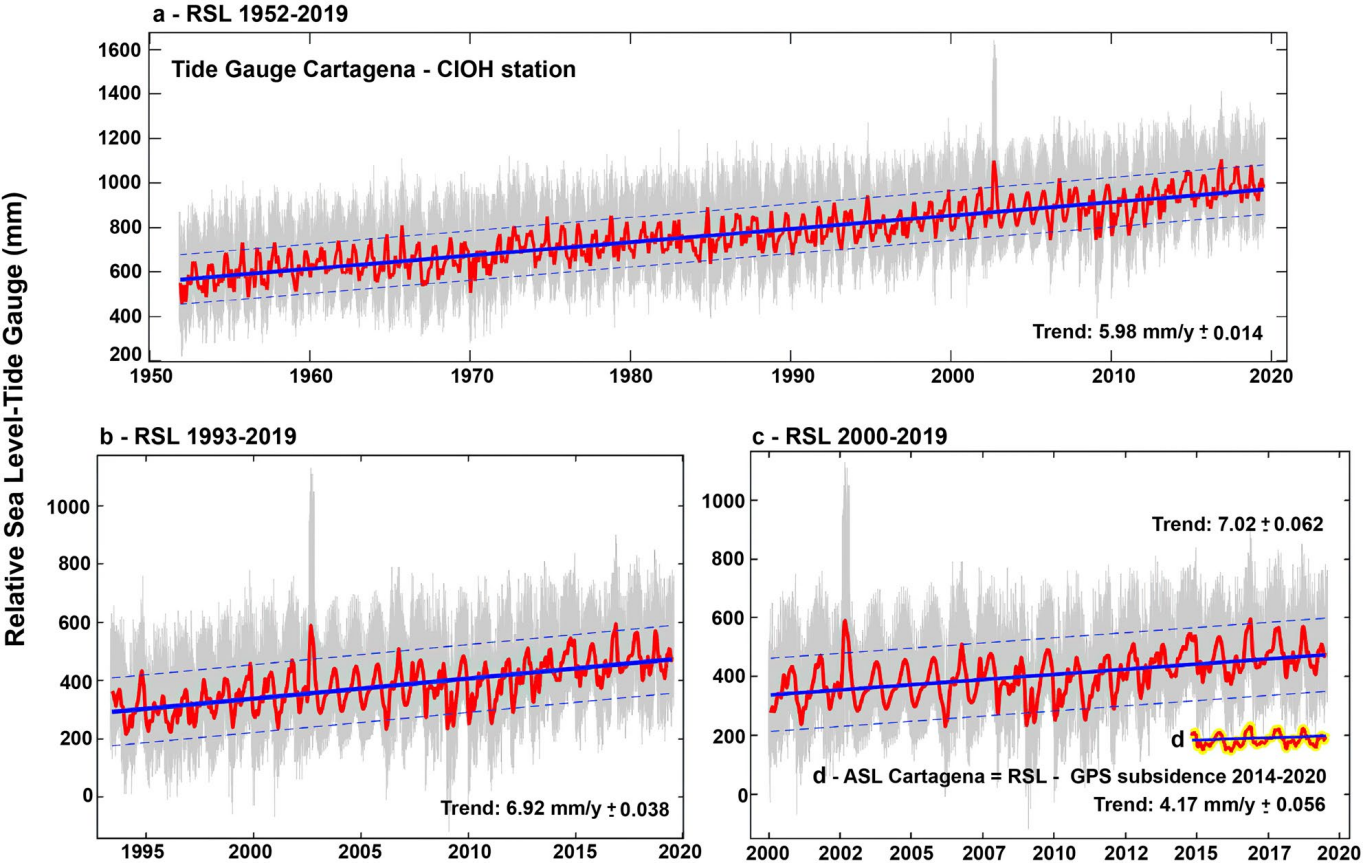
Time series of monthly relative sea level (RSL) data at the tide gauge in Cartagena for time spans 1952–2019 (**a**), 1993–2019 (**b**), and 2000–2019 (**c**), after being pre-processed to remove extremes and reconstruct gaps. (**d**) We include the absolute sea level trend (ASL) from 2014 to 2019 obtained after subtracting the GPS subsidence rate at the tide gauge. This new trend (4.17 ± 0.05 mm/yr) represents the steric component of RSL in Cartagena (**d**, red line with yellow highlight). We also show linear trends in RSL (blue) and data with a low-pass cosine-Lanczos filter and a cut-off period of 28 days (red) (see “Methods”). Data from the University of Hawaii Sea Level Center (UHSLC, https://uhslc.soest.hawaii.edu/) and the Oceanographic and Hydrographic Research Institute (CIOH) in Cartagena. Tide gauge plots generated by Matlab 2019b (https://www.mathworks.com/products/matlab.html).

Results revealed a long-term (> 60 years) rate of RSLR of 5.98 ± 0.01 mm/yr during the 1952–2019 period (Fig. 2a). During the last two decades, the RSLR has increased to a rate of 7.02 ± 0.06 mm/yr (Fig. 2b). This latter trend value of RSLR in Cartagena is more than twice the estimated ASLR rate for Cartagena’s offshore area (3.18 ± 0.29 mm/yr, shown in Supplementary Fig. S3). In other words, the RSL in Cartagena increased at much higher rates than the regional ASL over the last two decades. The differences between local RSL trends and global sea level rise are most likely due to climate variability controlling regional sea level changes at multi-decadal scales, vertical land motions, record length, instrumental failures, or a combination of all these factors^12^.

### Land subsidence derived from GPS geodetic stations

The linear trends of vertical land movements in Cartagena for the 2014–2020 period were derived from three GPS geodetic stations (BARU, CIOH, VCTG) that are part of the GeoRED Project (Geodesia: Red de Estudios de Deformación) (Fig. 1c), which is run by the Space Geodesy Research Group of the Colombian Geological Survey (CGS, Servicio Geológico Colombiano; formerly INGEOMINAS). Currently, the GeoRED network has 108 operating stations based on space geodesy technology located on the Nazca, South America and Caribbean plates^41^.

**Figure 3:**
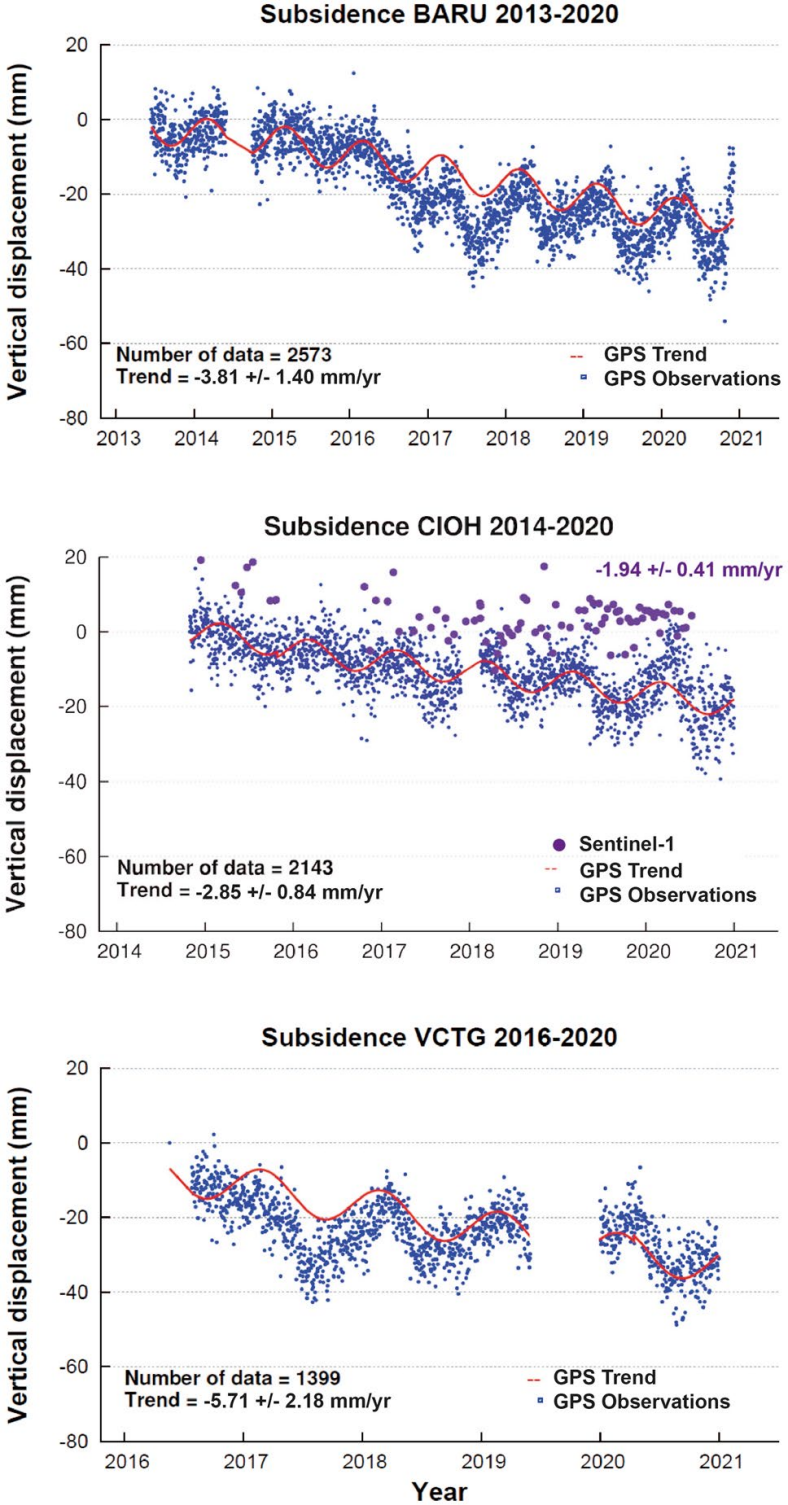
Time series (2013–2020) of subsidence (mm) at three GPS geodetic stations (Fig. 1c) in Cartagena (Data from the GeoRED Project, Space Geodesy Research Group of the Colombian Geological Survey-CGS, Servicio Geológico Colombiano). Sentinel-1 vertical displacement observations (mm) during the 2014–2020 period are plotted (purple) at the GPS-tide gauge station (CIOH) (center). GPS time series generated by GNUPLOT v 4.2 (http://www.gnuplot.info/).

Significant subsidence trends were observed in the coastal zone of Cartagena, including rates of -3.81 ± 1.40 mm/yr (2013–2020) at BARU station in the southwestern part of Cartagena Bay, and − 2.85 ± 0.84 mm/yr (2014–2020) at the CIOH station located near the tide gauge (Fig. 3). This value is similar to that obtained by the Nevada Geodetic Laboratory for the CART station, located in the same area, 60 m from the CIOH station. The VCTG station, located south of Cartagena near the Dique Canal (Fig. 1c), recorded a subsidence rate of − 5.71 ± 2.18 mm/yr (2016–2020) (Fig. 3).

We have identified the contribution of vertical land motions to RSLR on the coast of Cartagena using independent GPS geodetic values and assuming a linear rate of change. The subsidence rate of − 2.85 ± 0.84 mm/yr at the CIOH GPS station during the 2014–2020 period (Fig. 3) was extracted from the interannual RSL trend at the nearby tide gauge during the same period. The resulting mean ASL trend of 4.17 ± 0.05 mm/yr in Cartagena (Fig. 2d) indicates that subsidence-induced vertical motions represent 41% of the relative sea level rise observed in Cartagena. In other words, regional and global steric processes associated with global mean sea level rise only account for the remaining 59% of RSLR. The magnitude of the uncertainty associated with the contribution of subsidence to RSL can be explored by comparing tide gauge records and absolute mean sea level from altimetry data. In Cartagena, the 2000–2019 trend of RSLR of 7.02 ± 0.06 mm/yr (Fig. 2b) minus the satellite altimetry mean sea level trend of 3.18 ± 0.29 mm/yr (see Supplementary Fig. S3) results in an isostatic trend of − 3.84 mm/yr, a value which is close to the GPS subsidence rate of − 2.85 ± 0.84 mm/yr observed at the CIOH station (Fig. 3).

### Land subsidence with InSAR time-series observations

Until now, no land subsidence analysis has been carried on in the city of Cartagena. As a result, little is known about the spatial variability of subsidence in the city. The few studies have been restricted to using GPS technique and short time series of < 4.5 years^25–27,37^, however, there are no studies involving InSAR time-series analysis across the Cartagena region.

**Figure 4:**
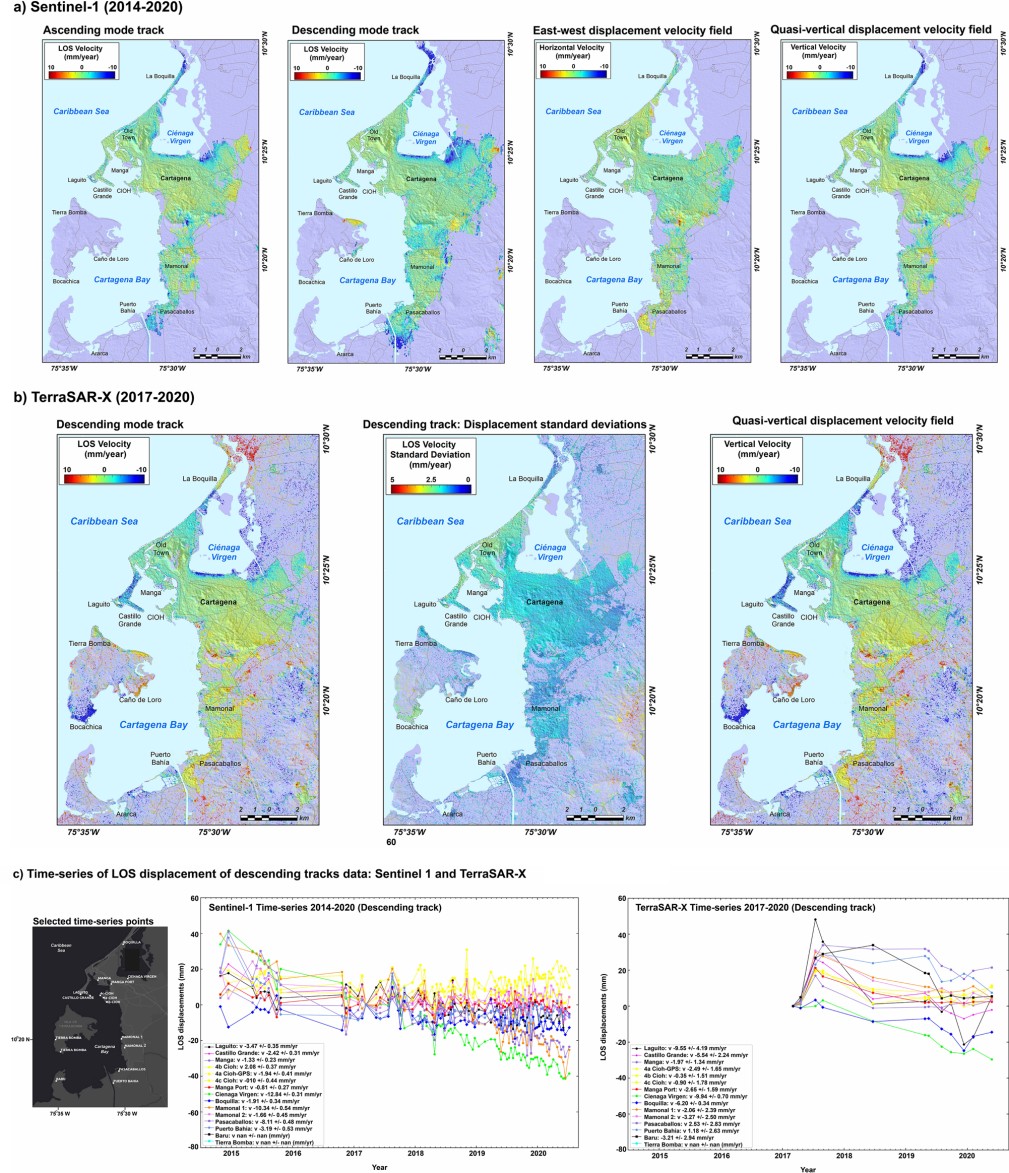
(**a**–**c**) Quantitative estimation of quasi-vertical displacements using Interferometric Synthetic Aperture Radar (InSAR) techniques. (**a**) Sentinel-1 images (2014–2020), showing ascending and descending mode tracks, and vertical and east–west displacement velocity fields. (**b**) TerraSAR-X image of descending mode track (left) and its standard deviations (center), and map of vertical displacement velocity field during the 2017–2020 period (right). (**c**) Time series of LOS displacements (mm/yr) at selected sites in the coastal zone of Cartagena. InSAR maps generated by open-source Python 3.8 using matplotlib module version 3.4.1.

We applied InSAR time-series analysis on Sentinel-1 and TerraSAR-X datasets obtained from 2014–2020 and 2017–2020, respectively, using Small Baseline approach^42,43^. The results are line-of-sight (LOS) InSAR time-series displacements and linear displacement rates maps, which were projected to quasi-vertical displacements to obtain subsidence maps of the city of Cartagena (Fig. 4; see “Methods” InSAR Data and Analysis).

Sentinel-1 time-series analysis reveals a widespread coastal subsidence processes in Cartagena (Fig. 4). Subsidence range in many coastal regions between − 1.66 mm/yr (Industrial zone) and -12.84 mm/yr (Cienaga Virgen). Also, the northern part of Cartagena Bay experiences important subsidence rates of 3.47 mm/yr and 2.42 mm/yr in Laguito and Castillo Grande, respectively. It is worth noting that GPS (2.85 ± 0.84 mm/yr) and Sentinel 1 (1.94 ± 0.41 mm/yr) subsidence values are of the same magnitude at the tide gauge station (CIOH) (Fig. 3; Supplementary Table S1). Sites at Mamonal, the industrial and port zone, reveal a wide range of cumulative subsidence rates between 7.1 mm and 16.2 mm. Southern coastal zones, including the oil port at Puerto Bahía, are exposed to cumulative subsidence values of up to 23.9 mm (Fig. 4; Supplementary Table S1).

TerraSAR-X satellite time-series analysis of data from 2017–2020 (Fig. 4) shows land subsidence rates of 9.5 mm/yr and 5.5 mm/yr at Laguito and Castillogrande, respectively. Estimates near the tide gauge site reveal a subsidence rate of 2.5 mm/yr, while the industrial sector of Mamonal has also incurred a subsidence rate of 3.3 mm/yr (see Supplementary Table S1).

### Sea level rise projections in Cartagena for 2050 and 2100

**Figure 5:**
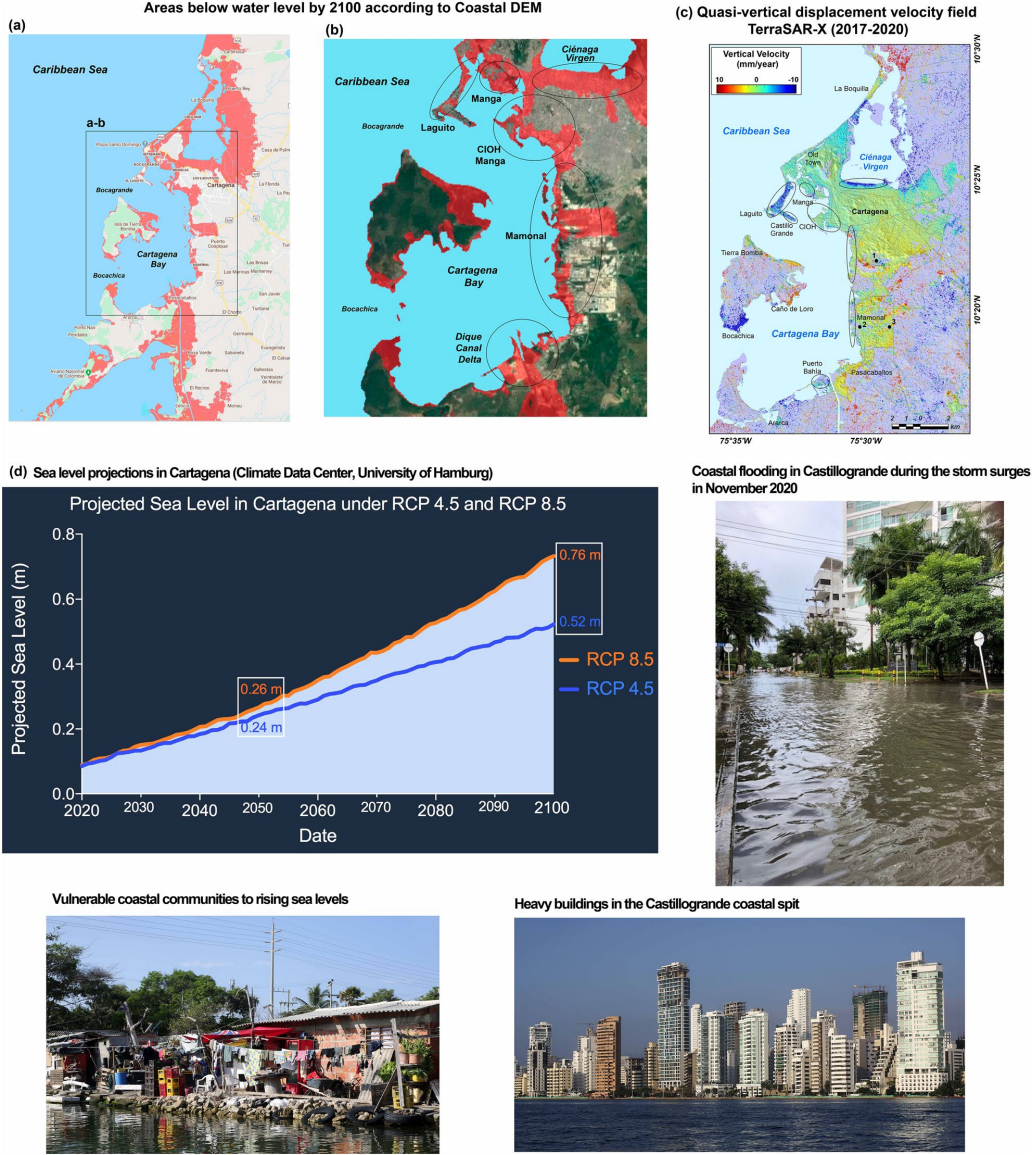
Areas of the Caribbean coast around Cartagena (**a**) and Cartagena Bay (**b**) below water level by the year 2100 based on a new digital elevation model, Coastal DEM, produced by Climate Central^32,44^ (DEMS from open source at https://climatecentral.org/pdfs/2019CoastalDEMReport ). (**c**) TerraSAR-X image of quasi-vertical displacements (mm/yr) in Cartagena during the 2017–2020 period (InSAR map generated by open-source Python 3.8 using matplotlib module version 3.4.1). Mud volcanoes El Rodeo (1), Membrillal (2), and Turbaco (3) are also shown. Most of current subsiding lands (**c**) coincide with projected flooded areas by 2100 (**b**). (**d**) Sea level projections in Cartagena by 2050 and 2100 obtained from the Integrated Climate Data Center of the University of Hamburg (ICDC, http://icdc.cen.uni-hamburg.de/1/daten/ocean/ar5-slr.html) (*Photo credits of Cartagena – Juan D. Restrepo, IDRC-BASIC Project)*.

Sea level projections in Cartagena for 2050 and 2100 were obtained from the Integrated Climate Data Center of the University of Hamburg (ICDC, http://icdc.cen.uni-hamburg.de/1/daten/ocean/ar5-slr.html). These data sets were acquired from the Intergovernmental Panel on Climate Change's (IPCC) Fifth Assessment Report (AR5) which projected global sea-level rise for 2100 based on different greenhouse gas (GHG) emission scenarios^29^. Also, we used a new digital elevation model, CoastalDEM, produced by Climate Central (https://coastal.climatecentral.org/) that uses neural networks to reduce the error of elevation data. This worldwide CoastalDEM shows that many of the world’s coastlines, including the Caribbean and Cartagena region (Fig. 5), are situated far lower in elevation than previously known from past DEMs^32,44^.

Gridded fields of projected sea level rise for the Cartagena region indicate that under the representative concentration pathway (RCP) of a moderate scenario of GHG reduction (RCP 4.5), sea level will rise 24 cm by 2050 and 52 cm by 2100. Under the unmitigated growth of emissions scenario (RCP 8.5), sea levels will rise 26 cm by 2050 and 76 cm by 2100 (Fig. 5). The CoastalDEM for Cartagena reveals that under the RCP 4.5 scenario, most parts of the coastline of Cartagena Bay, including Castillogrande coastal spit, Manga neighborhood, Manzanillo Island (CIOH tidal gauge site), and the industrial and port area of Mamonal, are likely to be flooded by 2100. In addition, the 2017–2020 TerraSAR-X satellite observations of vertical displacements largely coincide with the areas projected to be below water level by 2100 (Fig. 5).

## Discussion

The RSLR of 7.02 ± 0.06 mm/yr obtained during the 2000–2019 period (Fig. 2b) is about 25% higher than previous RSL trends estimated at the tide gauge of Cartagena, for example, 4.8 ± 0.6 mm/yr^24^ and 5.3 ± 0.3 mm/yr^20^, and more than twice the global mean ASLR of 2.9 mm/yr^29^. Other estimates of RSLR in Cartagena were also assessed in this study using water level data from pressure sensors installed in the southern part of Cartagena Bay (Fig. 1c). These data yielded estimates of RSLR trends for the 1952–2000 period which varied between 3.55 and 5.32 mm/yr (see Supplementary Fig. S4), values that are very similar to previous rates of RSLR^20,24^.

Subsidence rates resulting from vertical motions are two-fold greater than the rate of climate-driven sea level rise measured for the Caribbean Sea. GPS-derived subsidence trends obtained in this study, which range between − 5.70 ± 2.18 mm/yr and − 2.85 ± 0.84 mm/yr, are in agreement with previous estimates ^26,27,37^, and also with the results obtained by the Nevada Geodetic Laboratory. These previous subsidence estimates have low statistical confidence due to the short length of the time series, lack of data and large standard error values. In contrast, the bias and uncertainty of GPS velocity data are removed once time series are obtained for periods longer than 4.5 years^25,37,45^.

The time-series InSAR-derived subsidence rates in Cartagena for 2014–2020 with the high values up to 12.84 ± 0.31 mm/yr (Fig. 4; Supplementary Table S1), are the first available estimates of their kind to date providing spatial coverage of Cartagena. These subsidence rates are of the same magnitude as those estimated in densely urbanized areas of subsiding coastal and delta cities, such as Bangkok^46^, Jakarta^47^, Shanghai^48^, Lagos^11^, New Orleans^49,50^, and Miami^9^. Cartagena has become progressively urbanized during the last three decades, with a fast-growing expansion of buildings, hotels, and industrial areas. Although no published scientific data confirming that subsidence is enhanced by direct loading of the coastal land area by buildings, our Sentinel-1 InSAR-derived cumulative subsidence rate of 20.9 mm at Castillogrande may indicate that loading may be one of the factors associated with vertical motions. Evidence of Cartagena’s widespread subsidence has been revealed by the InSAR data and the city stands out as a hotspot of subsidence and rising sea levels across the whole Caribbean region.

The observed GPS (Fig. 3) and InSAR (Fig. 4) subsidence trends in Cartagena are in agreement with the presence of mud diapirism. Gravimetric and magnetic anomalies have identified diapiric bodies in the industrial zone as well as in the Dique delta^38,39^. Also, the southeast part of Cartagena is characterized by the presence of three active mud volcanoes, El Rodeo (see Supplementary Fig. S2), Membrillal, and Turbaco (Fig. 5c). In these volcanic regions, GPS temporal stations have measured subsidence trends ranging between 17 and 35 mm/yr^39^. Water extraction-induced subsidence has not been reported in Cartagena because there is no groundwater use in the city. In fact, water is provided to the public by Aguas de Cartagena, a company that chemically treats the water from the Canal del Dique.

Higher accuracy and higher resolution trends of relative sea level are likely important for improving coastal exposure assessments in the future. A recent study, employing a new digital elevation model (DEM), estimates a global total of 110 M people living on land below the current high tide line and 250 M on land below annual flood levels^32^. These numbers reveal that developed coastlines are three times more exposed to extreme coastal water levels than previously thought^32^. However, these projections of global vulnerability to sea level rise and coastal flooding assume a static coastal topography, without considering linear models of vertical land motion^32^. This limitation in global scenarios of coastal flooding using state-of-the-art DEMs indicates that flooding scenarios are actually even worse for coastal regions experiencing high subsidence rates.

The sea level and flood projections for Cartagena reveal that under a moderate scenario of GHG emissions (RCP 4.5), rising levels of 24 cm by 2050 and 52 cm by 2100 are expected (Fig. 5). These projections do not take into account subsidence rates or any other measurement of vertical land motion. Our GPS geodetic subsidence rate of − 2.85 ± 0.84 at the tide gauge site would imply additional increases of RSL of 8.3 cm by 2050 and 22.5 cm by 2100. Thus, conservative sea level projections for Cartagena must consider rising sea levels of up to 36 cm by 2050 and 85 cm by 2100. These RSLR estimates that include the effect of subsidence are at least 50% higher than projections based solely on GHG emissions, and so management plans that do not consider subsidence effects would be significantly underestimating the potential risk of future flooding. These findings indicate that cities like Cartagena must prepare themselves for much more difficult futures than the projections anticipated in global assessments.

Another environmental issue faced by Cartagena and its bay is the future sediment budget and its impact on RSLR trends. The Magdalena River, the main contributor of continental fluxes into the Caribbean Sea, delivers important amounts of water and sediments into Cartagena Bay through the Dique Canal, a man-made distributary channel. During the last three decades, the Dique Canal has discharged ~ 177 Mt of sediment to the coastal zone, of which 52 Mt was discharged into Cartagena Bay. Currently, the canal drains 6.5% and transports 5.1% of the Magdalena’s water discharge and sediment load, respectively^51^. The canal has formed a fast protruding delta lobe inside Cartagena Bay (Fig. 1). Due to the large amounts of sediment fluxes into the bay, a process that has changed a coral reef crystalline bay into a turbid fluvial estuary, the Colombian government is planning a huge hydraulic intervention in the canal with the intention of retaining the sediment load flowing into Cartagena Bay. However, it has been proven globally that a consequence of reduced sediment delivery or deposition is the under-compensation of land subsidence and increased rates of RSLR^5^.

Future sea level scenarios in the Magdalena delta, with a 50% reduction in sediment flux due to reservoir construction, would lead to an increase in RSLR from 3.3 to 7.8 mm/yr^5^. An expanded reservoir in the Magdalena would have approximately the same impact on RSLR rates as that of transitioning from RCP 2.6 to 8.5^5^. Thus, the projections for the Cartagena RSLR trend under a 100% sediment retention scenario could more than double the current RSLR of 7.02 mm/yr due to the imbalance of sediment flux for compensating subsidence rates.

Freshwater runoff from the Magdalena River via the Dique Canal has also been shown to have an impact on the seasonal and spatial variability of sea level in Cartagena Bay. Hydrodynamic simulations using the MOHID Water Modelling System, calibrated in Cartagena Bay with a high-resolution 3D configuration, have demonstrated how water levels can vary in the bay due to freshwater accumulation and wind-driven “pile-up”^52^. During the windy season (Jan.-Mar.), strong northerly winds generate a north–south gradient with mean water levels up to 12 mm higher at the southern end of the bay than at the northern end, while during the transitional (April-July) and rainy (Aug.-Nov.) seasons, mean water levels are heightened by approximately 4–5 mm in the central part of the bay near the Mamonal industrial zone due to the accumulation of freshwater discharge from the Dique Canal. Overall, the bay’s mean water level is approximately 10 cm higher in the rainy season than in the transitional season^52^, in agreement with previous research on the seasonal cycle of water levels in the Caribbean^53^.

Recently, it has been documented that the rise in sea levels caused by climate change will result in storm surges, extreme high tides, and wave setup pushing water farther inland^54^. Projections of global-scale extreme sea levels (ESL) and resulting episodic coastal flooding analyses show that under a mean RCP 8.5 scenario, there will be a 48% increase of the world’s land area at risk of flooding by 2100. A total of 68% of the flooded global coastal area will be caused by tide and storm events, while 32% would be due to projected regional sea level rise^54^. In this global assessment of ESL over the twenty-first century, Cartagena is one of the four Caribbean inundation hotspots with future ESL in the range of 0.5–1.5 m by 2100^54,55^. Although the focus of the general public in Colombia often tends to be on the rate and magnitude of sea level rise due to climate change, Cartagena also faces major threats of coastal flooding and erosion due to land subsidence and extreme oceanographic conditions.

Coastal communities in Cartagena are especially vulnerable to rising sea levels because the coastal morphology and infrastructure are adapted for small sea level variations^20^. We warn that future city planning, including the conservation of cultural heritage, flood mitigation, and infrastructure development, must implement consistent subsidence measurements and modeling across the city in order to link science with socioeconomic implications. For policy makers in Cartagena, politically tough decisions lie ahead. What do they conserve on the water’s edge? How do they reimagine the city in the century of rising sea levels? The biggest challenge will be getting Cartagena’s society to understand, cope with, and plan for sea level rise, and then to discuss the trade-offs of mitigation options.

## Methods

### Relative sea level (RSL)

Monthly records of RSL for the 1952–2000 period at the tide gauge in Cartagena (Fig. 1c) were obtained from the University of Hawaii Sea Level Center (UHSLC, https://uhslc.soest.hawaii.edu/)^55^. Further hourly records for the 2001–2019 period were obtained from the Oceanographic and Hydrographic Research Institute (CIOH) in Cartagena. We performed harmonic analyses on the CIOH water level data to calculate the amplitude and phase of tidal constituents, and the main tidal statistics. The data were approximately 68% complete and missing records were interpolated with harmonic analysis. Monthly averaged values were calculated from daily values following the procedure detailed by the Permanent Service for Mean Sea Level (PSMSL; www.psmsl.org/data/obtaining/). The RSLR trend was estimated by a least squares linear fitting from the tide gauge records over the total 1952–2019 period. The length of the tide gauge records (> 60 years) allows for an estimation of long-term rates of RSL by minimizing the impact of the inter-annual and decal signals.

The seasonal variability was removed from the RSLR time series by subtracting the climatological monthly mean from the monthly values. Uncertainties were defined as the Standard Error (SE) of the fit adjusted for lag-1 autocorrelation^12^. Spurious data extremes were screened by the phase-space thresholding method^56^ and gaps were reconstructed based on harmonic analysis from a least-squares fit of tidal harmonics K_1_, M_2_, O_1_, N_2_, and S_2_^57^. We also displayed RSL data with a low-pass cosine-Lanczos filter^58^ and a cut-off period of 28 days (Fig. 2). Further observations of relative sea level trends from pressure sensors in Cartagena Bay are shown in Supplementary Information (see Supplementary Fig. S4).

### Satellite altimetry data of absolute sea level (ASL) across the Caribbean offshore area of Colombia

Monthly ASL data during the 1993–2015 period were obtained from AVISO^40^ (Archiving, Validation and Interpretation of Satellite Oceanographic data, http://www.aviso.altimetry.fr) at five stations along the Caribbean coast of Colombia, including an offshore site near Cartagena (see Supplementary Fig. S3). The AVISO ASL datasets consist of merged multi-mission data (TOPEX/Poseidon, Jason-1, Jason-2) between 1993 and 2015. The horizontal resolution for the AVISO data is 1/4°. AVISO ASL trends in the Caribbean were estimated by least squares linear fitting for comparison with tide gauge data at Cartagena. Also, the global mean sea level trend for 1993–2019 was obtained from the same AVISO source (Supplementary Fig. S3).

### Subsidence trends from GPS geodetic records

The linear trend magnitudes of vertical land movements in Cartagena for the 2013–2020 period were derived from GPS geodetic stations from the GeoRED Project (Geodesia: Red de Estudios de Deformación), which is run by the Space Geodesy Research Group of the Colombian Geological Survey (CGS, Servicio Geológico Colombiano; formerly INGEOMINAS). This precise vertical motion field is based on three permanent stations in Cartagena (Fig. 1c) with a minimum of 2.5 years of observations^41,45^.

All GPS data were processed with processed with GIPSY-X/RTGx (GNSS-Inferred Positioning System and Orbit Analysis Simulation Software) v 1.3 developed by the Jet Propulsion Laboratory (JPL), California Institute of Technology^59,60^. Daily station coordinates are expressed in ITRF2014. The station velocities were computed using the HECTOR software^60^, developed at SEGAL (Space & Earth Geodetic Analysis Laboratory at the University of Beira Interior, Portugal) that is used to estimate the linear trend in time-series with temporal correlated noise.

A power-law plus white noise model was assumed. For each time series, a power-spectrum plot was generated from the residuals, and the predicted power-spectrum of the noise model was compared with the observed power spectrum to verify that the correct noise model had been properly applied. Seasonal signals, including annual and semi-annual signals, were included in the estimation of the secular velocities in order to reduce their influence on the estimated velocities^41^. We follow the current state-of-art approach that assumes that the amplitude of such signals is constant during each considered period and described by a sinusoidal curve. It has been demonstrated^61^ that when the time series are longer than 3–5 years, the remaining influence of the seasonal signals on the estimated trend can be neglected^41^.

The ASL and the contribution of vertical land motion (subsidence) to the RSLR (Fig. 2c) at the tide gauge in Cartagena were computed following the Eq. (1)^27^:

1 $${\text{T}}_{{{\text{ASL}}}} = {\text{ T}}_{{{\text{RSL}}}} + {\text{ T}}_{{{\text{VLM}}}}$$

where T_ASL_ is the absolute sea level trend (geocentric), T_RSL_ is the relative sea level trend calculated from the tide gauge data, and T_VLM_ is the estimated subsidence trend of the nearest GPS station, which for Cartagena, is located next to the tide gauge at CIOH (Fig. 1c).

### Subsidence rates from time-series InSAR data

We used Sentinel-1 (94 scenes of ascending and 64 scenes of descending orbit track) and TerraSAR-X (12 scenes of descending orbit track) acquisitions to form a Small-Baseline (SB) network of unwrapped interferograms covering the period of 2014–2020 and 2017–2020, respectively (Fig. 4; Supplementary Fig. S5). The unwrapped interferograms were generated with the InSAR Scientific Computing Environment (ISCE) software^43,62^, including 285 and 249 interferograms from Sentinel-1 ascending and descending orbit track datasets, respectively, and 13 interferograms from TerraSAR-X descending orbit track. The Shuttle Radar Topography Mission 1-arc sec (SRTM-1) Digital Elevation Model^63^ was used to remove tropospheric phase and perform geocoding of unwrapped interferograms to the WGS84 reference frame. The phase unwrapping was done using the minimum cost-flow Statistical-Cost, Network-Flow Algorithm for Phase Unwrapping (SNAPHU) algorithm^64^. We selected the spatial unwrapping reference point at location with latitude of 10.429° and longitude − 75.525°, which we considered presumably stable, i.e. not affected by any surface deformation.

We used the Miami InSAR time-series software in Python (Mintpy)^65^ and Generic InSAR Analysis Toolbox^66,67^ for the InSAR time-series analysis on Sentinel-1 and TerraSAR-X interferogram stacks, respectively. The Sentinel-1 SB networks are formed with the interferograms that have an average spatial coherence higher than 0.42 in the defined area of interest (10.27° S–10.5° N, − 75.57° E–75.46° W). Also, we excluded the interferograms with unwrapping phase errors. After the SB inversion of interferograms networks for the line-of-sight (LOS) time-series displacements, we applied a tropospheric phase delay correction using ERA-5 reanalysis model^68^, linear de-ramping to remove a long-spatial wavelength phase signal, and topographic phase residual correction to compensate for the DEM error^69^. We masked all points with temporal coherence lower than 0.7 and estimated LOS linear displacement rates from corrected time-series displacements. The SB TerraSAR-X network consisted of interferograms with shorter temporal baselines to maintain the overall good average spatial coherence over the study area. We inverted the TerraSAR-X interferograms network using the New Small Baseline Subset (NSBAS)^70^ algorithm with additional orbital de-ramping correction based on GPS displacements on 14 stations located in the region. After the NSBAS inversion, we estimated a linear LOS displacement rates (Fig. 4; Supplementary Fig. S5) from the obtained time-series displacements.

The InSAR subsidence (vertical displacement) maps of the Cartagena city (Fig. 4; Supplementary Fig. S5) were obtained with the projection of LOS InSAR time-series displacement rates into the quasi-vertical and horizontal displacement directions. The Sentinel-1 subsidence map was obtained using the LOS decomposition of the displacement rate on the same point acquired from a different Sentinel-1 viewing directions (i.e., ascending and descending orbit track Sentinel-1 acquisitions)^71^. Due to low InSAR sensitivity to horizontal north–south surface movements, we assumed no horizontal movements in this direction and projected the LOS displacement rates into the vertical and horizontal east–west displacement rates for 2014–2020. As we do not have both viewing TerraSAR-X acquisitions, we projected the LOS displacement rates for the 2017–2020 period to quasi-vertical ones with an assumption of no horizontal movements, by dividing the LOS displacement rates with the cosine of the satellite acquisition incidence angle. The three displacement vector components of LOS displacement per each dataset is shown in Eq. (2):

2 $$\left[ {\begin{array}{*{20}l} {Sentinel1_{LOS} ._{asc} } \hfill \\ {Sentinel1_{LOS} ._{dsc} } \hfill \\ {TerraSAR - X_{LOS} ._{dsc} } \hfill \\ \end{array} } \right] = \left[ {\begin{array}{*{20}l} { - 0.132} \hfill & { - 0.617} \hfill & {0.776} \hfill \\ { - 0.133} \hfill & {0.620} \hfill & {0.773} \hfill \\ { - 0.112} \hfill & {0.567} \hfill & {0.816} \hfill \\ \end{array} } \right]*\left[ {\begin{array}{*{20}l} {U_{NS} } \hfill \\ {U_{EW} } \hfill \\ {U_{V} } \hfill \\ \end{array} } \right]$$

where [U_NS_, U_EW_, U_V_] represent displacements in horizontal North–South, East–West, and Vertical direction, respectively.

### Sea level and flood projections

We followed the Intergovernmental Panel on Climate Change's (IPCC) Fifth Assessment Report (AR5) projected global sea-level rise by 2100, forced by different GHG emission scenarios^29^. Projected rise of sea level under each scenario is based on the addition of specific forces including steric changes, melting of glaciers and ice caps, the Greenland Ice Sheet, the Antarctic Ice Sheet, and land water storage^12^. Sea level projections in Cartagena for 2050 and 2100 were obtained from the Integrated Climate Data Center of the University of Hamburg (ICDC, http://icdc.cen.uni-hamburg.de/1/daten/ocean/ar5-slr.html).

We used two GHG scenarios (RCP) from moderate (RCP 4.5) to unmitigated growth of emissions (RCP 8.5). The data consist of gridded fields of projected sea-level change estimated as the 20-yr mean differences between the 2081–2100 and the 1986–2005 periods, with a horizontal resolution of 1°. The RCP 4.5 and RCP 8.5 scenarios were assessed for the Cartagena region from data produced by the ICDC-University of Hamburg (Fig. 5). Also, we used a new digital elevation model, CoastalDEM, produced by Climate Central that uses neural networks to reduce the error of elevation data. This worldwide CoastalDEM shows that many of the world’s coastlines, including the Caribbean and Cartagena region (Fig. 5), are situated far lower in elevation than previously known from past DEMs^22,32^.

## Supplementary Information

Supplementary Information.

## Data Availability

The data availability is outlined in “Methods” section. Correspondence and requests for materials should be addressed to J.D.R.A, H.M, and MG.
