# Supplementary material for: Coastal subsidence increases vulnerability to sea level rise over twenty first century in Cartagena, Caribbean Colombia
Source: Sci Rep. 2021 Sep 23;11:18873. doi: 10.1038/s41598-021-98428-4 (PMC8460661; doi:10.1038/s41598-021-98428-4)
Supplement: Supplementary file 1 — Supplementary Information. [file 41598_2021_98428_MOESM1_ESM.pdf]

Supplementary Information for

Coastal subsidence increases vulnerability to sea level rise over 21st Century in Cartagena, Caribbean Colombia

Juan D. Restrepo-Angel[1,*], Héctor Mora-Páez[2], Freddy Díaz[2], Marin Govorcin[3], Shimon Wdowinski[4], Leidy Giraldo-Londoño[2], Marko Tosic[1], Irene Fernández[1], Juan F. Paniagua-Arroyave[1], and José F. Duque-Trujillo[1]

[1]Universidad EAFIT, Department of Earth Sciences, School of Sciences, Medellín, AA 3300, Colombia
[2]Colombian Geological Survey, Space Geodesy Research Group, Bogotá, Colombia
[3]Institute of Geomatics, Faculty of Geodesy, University of Zagreb, Croatia
[4]Institute of Environment, Department of Earth and Environment, Florida International University, Miami
*jdrestre@eafit.edu.co

Mud diapirism and tectonics in the Cartagena region. Mud intrusion (diapirism) and volcanism are characterized by fluid-rich and fine-grained sediments that ascend within a lithological succession due to their buoyancy. These geologic features and processes have long been associated with the occurrence of petroleum, gas, regional volcanic and earthquake activity, and collisional tectonic settings. Most importantly, mud volcanoes occur along convergent plate margins or collisional settings where fluid-rich sediment is accumulated in deep-sea trenches at high rates. Such deposits then enter the subduction factory, where liquids and volatiles are released due to compactional stress and temperature. After these fluids are released, sediment compaction and further subsidence occur[1].

In the Cartagena region, early descriptions of these mud volcanoes date back from the 1960s[2,3]. Later, other geologic assessments classified the coral reef islands around Cartagena, including Tierra Bomba island (Supplementary Fig. S1b), as diapiric domes due to upheaval mechanisms during the late Holocene[4-8]. Based on recent seismic profiles[9] (Supplementary Fig. S1c) and studies[10-13], many diapiric formations and domes have been mapped in Cartagena Bay[14] (Supplementary Fig. S1b), showing that mud diapirism-induced subsidence in Cartagena is much greater than previously thought. Approximately more than 45 mud domes, hereafter pop marks, have been identified in the bottom of the bay (Supplementary Fig. S1b).

Further evidence of recent tectonism in the Cartagena region includes the upheaval of coastal terraces at Tierra Bomba at a rate of 3.11 mm/yr, the upheaval of a diapiric dome with a Pleistocene reef, locally known as La Popa Hill [8], and different local tectonic faults like the Dique fault (Supplementary Fig. S1b), an important component of the neotectonic activity in the Cartagena region[4]. Also, many mud-volcanoes have had eruptions during recent times in Cartagena. For example, a major event in the El Rodeo Volcano occurred in May 2014. This event produced major structural damages in buildings as well as land subsidence in the area (Supplementary Fig. S2). There is no doubt that the complex geology of Cartagena, which is characterized by compressional and transpressional tectonics, active diapirism and mud volcanism, is a key factor affecting the vertical land motions observed in the coastal bay and city.

Satellite altimetry data of absolute sea level (ASL) across the Caribbean offshore area of Colombia. Significant trends and values similar to the absolute global mean sea level rate (2.9 ± 0.1 mm/yr)[14] were found over the region covering the northern and southern parts of the Caribbean offshore area. Overall, the whole Caribbean marine area of Colombia has witnessed an increasing mean ASL trend of 2.96 ± 0.12 mm/yr during the 1993-2015 period. Trends of ASL rise range from 2.83 ± 0.18 mm/yr in the northern region of the Guajira Peninsula, to 3.22 ± 0.22 mm/yr in the offshore area of Cartagena (Supplementary Fig. S3).

Relative sea level observations from pressure sensors. To compare and contrast the relative sea level trends between the tide gauge and the southern part of Cartagena Bay, we made water level observations at four stations (Fig. 1c, Supplementary Fig. S4). Time series measurements of water level elevation were obtained using several near-bottom pressure sensors. The instruments were deployed at the bottom of each station, programmed to calculate a 2-min average every 10 min, and remained moored from April to October 2013.

Hourly relative sea level (RSL) data from the tide gauge at CIOH (Fig. 1c) during the same period of April-October 2013 were obtained from the Hydrological and Environmental Institute of Colombia (IDEAM), which was previously the institution in charge of the tide gauge. These RSL tide gauge data were related to water level observations from the pressure sensor stations through linear fit-transfer equations. To obtain the long-term RSL trend for 1952-2000 at the location of each pressure sensor (Supplementary Fig S4a), each transfer equation was applied to the RSL series for the 1952-2000 period. Overall, the long-term RSLR trends for 1952-2000 in the southern part of the bay varied between 3.55 and 5.32 mm/yr (Supplementary Fig. S4), values that are very similar to previous rates of RSLR[15,16].

Supplementary References

1. Kopf, A.J. Significance of mud volcanism. *Reviews of Geophysics*. **40**, 1005 (2002).
2. Ganser, A. U¨ ber Schlammvulkane und Salzdome, Vierteljahresschr. *Naturforsch. Ges. Zuerich*. **105**, 1-46 (1960).
3. Toto, E.A. & Kellogg, J.N. Structure of the Sinu-San Jacinto fold belt-An active accretionary prism in northern Colombia. *J. S. Am. Earth Sci*. **5**, 221–222 (1992).
4. Ordoñez, J.C. Controle neotectonico de diapirismo de lama na regiao de Cartagena, Colombia. Master Science Thesis, Universidad Federal Fluminense, Área de Geología y Geofísica marina, Brasil, 2008 (unpublished).
5. Page, W. Holocene deformation of the Caribbean coast, northwestern Colombia in *Field Trip C: General Geology, Geomorphology and Neotectonics of Northwestern Colombia 10th Caribbean Geol. Conference, Cartagena* (eds. Duque-Caro, H. *et al.*) 1-20 (Ingeominas, 1983).
6. Duque-Caro, H. Structural style, diapirism and accretionary episodes of the Sinú-San Jacinto terrane, southwestern Caribbean borderland. *Geological Society of America, Memoire.* **162**, 303–316 (1984).
7. Vernette, G., Mauffret, A., Bobier, C., Briceño, I. & Gayet, J. Mud diapirism, fan sedimentation and strike-slip faulting, Caribbean Colombian Margin. *Tectonophysics*. **202**, 335-349 (1992).
8. Martínez, J.I. *et al.* Late Holocene marine terraces of the Cartagena region, southern Caribbean: The product of neotectonism or a former high stand in sea level. *Journal of South American Earth Sciences.* **29**, 214-224 (2010).
9. Restrepo, J.D. & Correa, I.D. Environmental Assessment of the Barú Island (Technical Report, Universidad EAFIT-Ecoral, 2015).
10. Andrade, C.A., Ferrero-Ronquillo, A.J., León-Rincón, H., Mora-Páez, H. & Carvajal, J. H. Sobre cambios en la línea de costa entre 1735 y 2011 y la subsidencia en la Bahía de Cartagena de Indias, Colombia. *Rev. Acad. Colomb. Cienc. Ex. Fís. Nat.* **158**, 94-106 (2017).
11. Carvajal, J.H. Mud diapirism in the Central Colombian Caribbean Coastal zone in *Landscapes and Landforms of Colombia* (ed. Hermelin, M.) 35-53 (Springer, 2016).
12. Dill, H.G. & Kaufhold, S. The Totumo mud volcano and its near-shore marine sedimentological setting (North Colombia) – From sedimentary volcanism to epithermal mineralization. *Sedimentary Geology*. **366**, 14-31 (2018).

13. Carvajal, J.H. & Mendivelso, D. Catálogo de Volcanes de lodo, Caribe Central colombiano (Technical Report, Colombian Geological Survey, Bogotá, 2017).
14. Church, J. A. *et al.* Climate change 2013: The Physical Science Basis. Contribution of Working Group I to the Fifth Assessment Report of the Intergovernmental Panel on Climate Change. *Cambridge University Press, New York* **2013**, 1137-1216 (2013).
15. Torres, R. & Tsimplis, M.N. Sea-level trends and interannual variability in the Caribbean Sea. *J. Geophys. Res. Oceans.* **118**, 2934-2947 (2013).
16. Andrade, C.A. Cambios recientes del nivel del mar en Colombia in *Deltas de Colombia: morfodinámica y vulnerabilidad ante el Cambio Global* (ed. Restrepo, J.D.) 101-121 (Fondo Editorial Universidad EAFIT, 2008).
17. H. Mora-Páez, J.H. Carvajal, A. Ferrero, H. León, C.A. Andrade, On natural gas venting and preliminary results on subsidence in Cartagena bay (Colombia). *Boletín Científico CIOH.* **37**, 35-51 (2018).
18. Aviso. MSS CNES-CLS15, https://www.aviso.altimetry.fr/en/data/products/auxiliary-products/mss.html (2015).
19. Tosic, M., Martins, F., Lonin, S., Izquierdo, A. & Restrepo, J.D. Hydrodynamic modelling of a polluted tropical bay: Assessment of anthropogenic impacts on freshwater runoff and estuarine water renewal. *Journal of Environmental Management.* **236**, 695-714 (2019).

Supplementary Figure S1: (**a**) Bathymetry and major geologic features of Cartagena's offshore area. (**b**) Geologic setting of the Cartagena region showing location of tectonic faults, mud diapirs on land and submarine pop marks (location of pop marks based on[17]). Maps generated by ArcMap from ESRI, https://desktop.arcgis.com/es/arcmap/

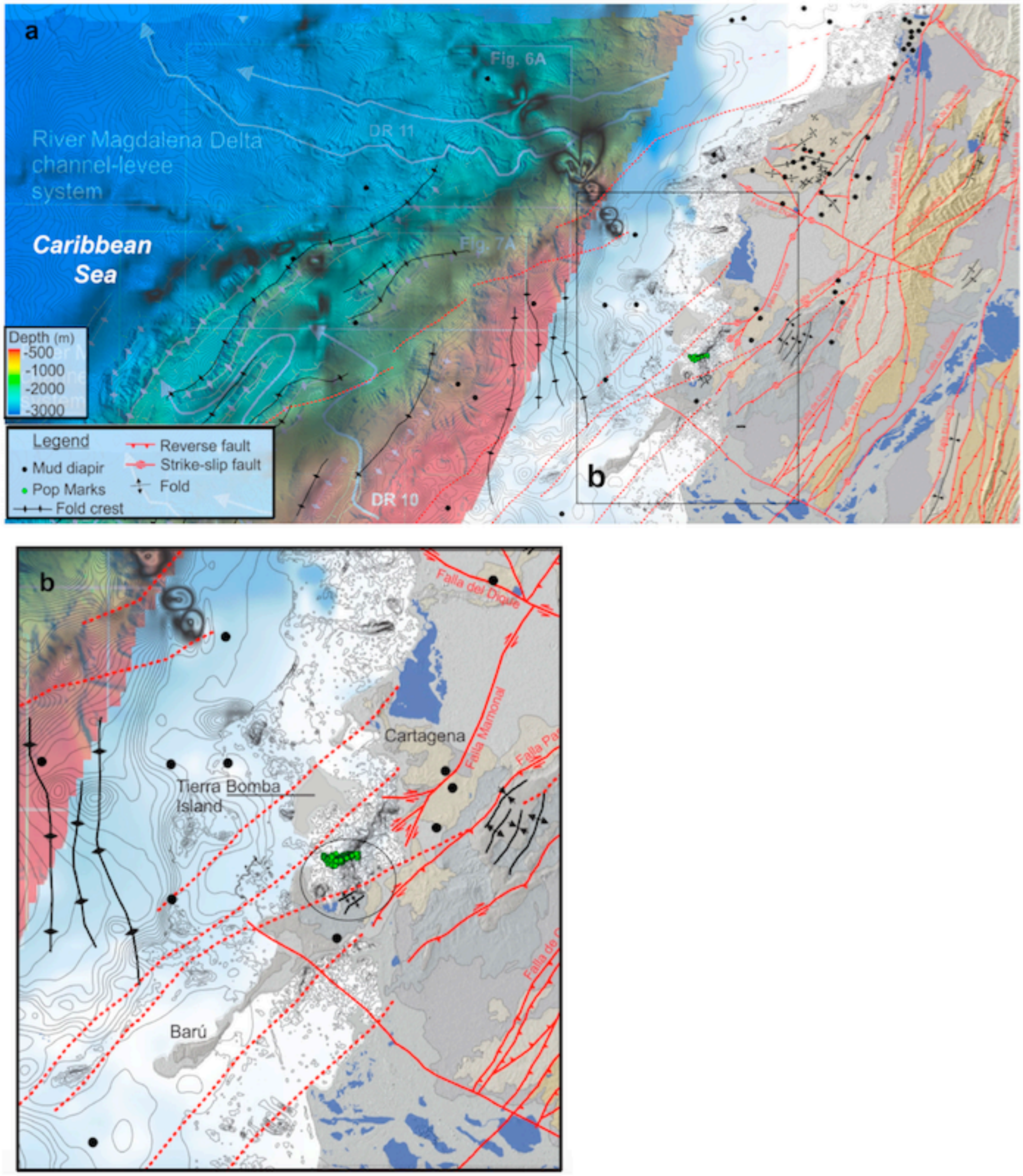

Supplementary Figure S2: Photos of the El Rodeo mud volcano in the Technological University of Bolívar campus during an eruption in 2014. Similar volcanoes and mud diapirs are present in the marine bottoms of Cartagena Bay and offshore (*Photo credits of El Rodeo volcano: Héctor Mora-Páez*).

MUD VOLCANO EL RODEO LOCATED IN CARTAGENA

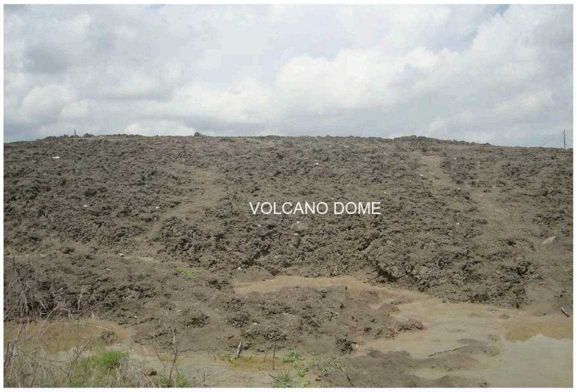

MUD ERUPTION OF EL RODEAO VOLCANO IN 2014

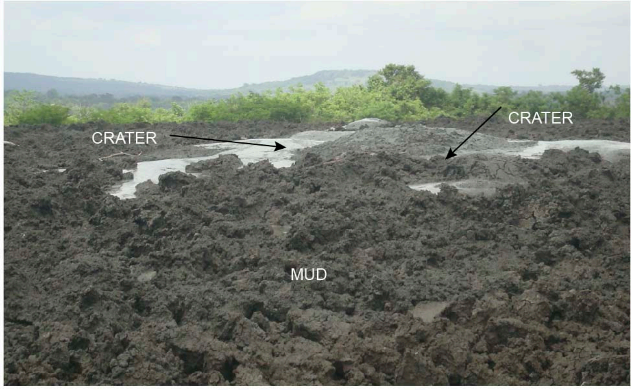

STRUCTURAL DAMAGES IN BUILDINGS 2014

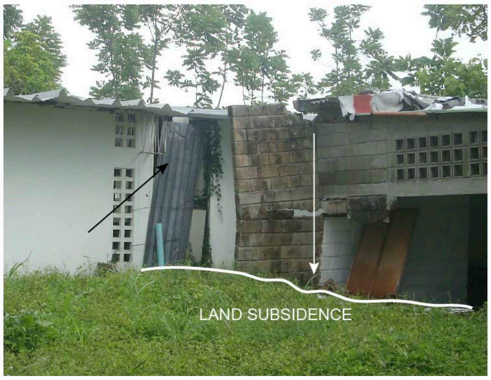

STRUCTURAL DAMAGES IN BUILDINGS 2014

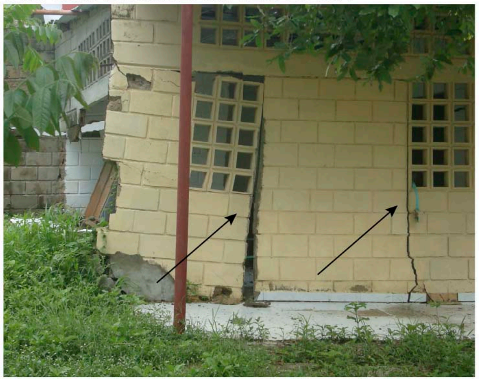

Supplementary Figure S3: (**a**) Global map of mean sea level trends from satellite altimetry data, 1993-2019. (**b**) Offshore Colombian Caribbean stations of altimetry data from 1993-2015 analyzed in this study. (**c**) Time series of mean sea level (1993-2015) at four offshore stations, including the Cartagena region, obtained from AVISO[18] (Archiving, Validation and Interpretation of Satellite Oceanographic data, http://www.aviso.altimetry.fr). Sea level plots generated by Matlab 2019b (https://www.mathworks.com/products/matlab.html).

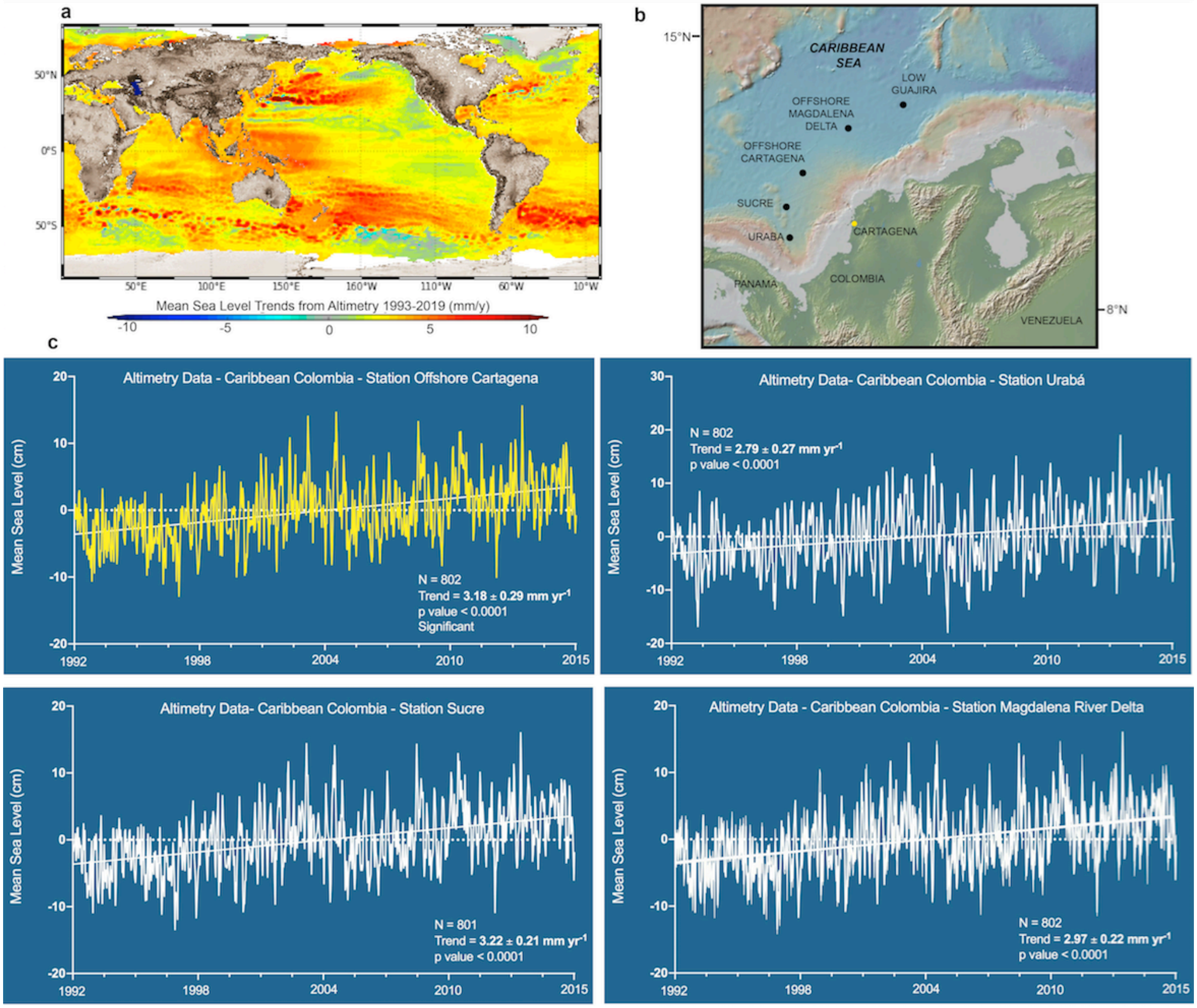

Supplementary Figure S4: (**a**) Map of Cartagena Bay showing the stations of water level observations using near-bottom pressure sensors. (**b-d**) Linear fit of relative sea level trends for 1952-2000 at three stations in the southern part of Cartagena Bay. (**e**) Relative sea level for 1952-2000 at the internal bay station from near-bottom pressure sensor data. Sea level dispersion and series plots generated by Matlab 2019b (https://www.mathworks.com/products/matlab.html).

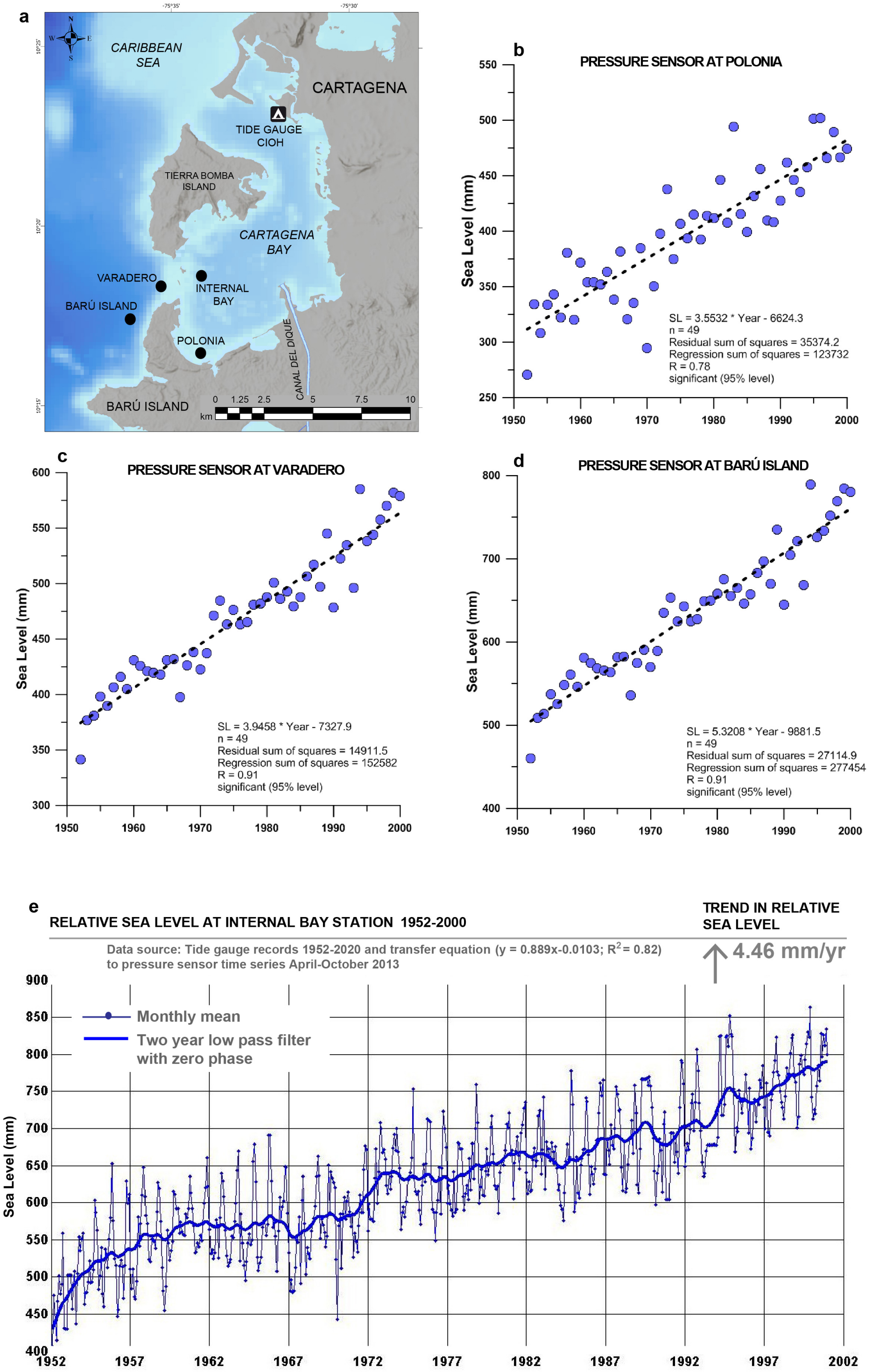

Supplementary Figure S5: (a) Sentinel-1 images of LOS velocity standard deviations for ascending and descending mode tracks. Maps of vertical and horizontal cumulative displacements (cm) during the 2014-2020 period are also shown. **(b)** Terrasar X map of vertical cumulative displacements (cm) during the 2017-2020 period. InSAR maps generated by open-source Python 3.8 using matplotlib module version 3.4.1.

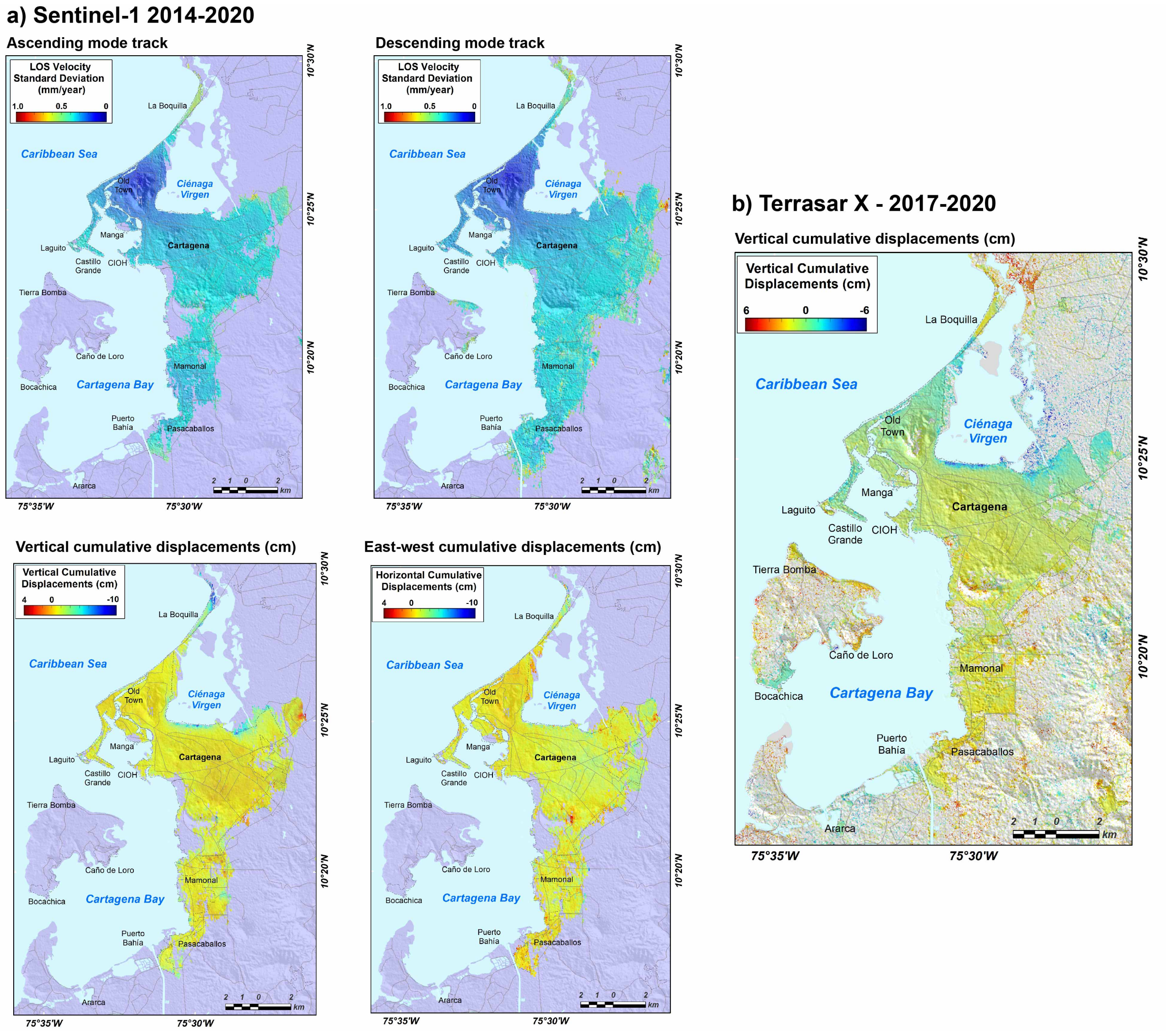

Site Cartagena Bay	*Radar sensor*	*Location (Lat-Long)*	*Cumulative Subsidence (mm)*	*Subsidence Velocity ± Standard deviation (mm/yr)*
Laguito	Sentinel-1	10.39.37 -75.55.93	**-21.25**	**-3.47±0.35**
	Terrasar-X	10.39.37 -75.55.93	-5.05	-9.55±4.19
Castillo Grande	Sentinel 1	10.39.24 -75.54.82	**-20.99**	**-2.42±0.31**
	Terrasar X	10.39.24 -75.54.82	-2.07	-5.54±2.24
Manga	Sentinel 1	10.41.19 -75.53.83	**-10.94**	**-1.33±0.23**
	Terrasar X	10.41.19 -75.53.83	3.03	-1.97±1.34
4a-CIOH-GPS	Sentinel 1	10.38.98 -75.53.15	**-16.4**	**-1.94±0.41**
	Terrasar X	10.38.98 -75.53.15	5.03	-2.49±1.65
4c-CIOH-Norh	Sentinel 1	10.38.46 -75.52.78	**-8.43**	-0.10±0.44
	Terrasar X	10.38.46 -75.52.78	11.31	-0.90±1.78
Manga-Port	Sentinel 1	10.40.71 -75.52.19	**-7.17**	**-0.81±0.27**
	Terrasar X	10.40.71 -75.52.19	2.38	-2.65±1.59
Ciénaga Virgen	Sentinel 1	10.41.39 -75.50.16	**-72.32**	**-12.84±0.31**
	Terrasar X	10.41.39 -75.50.16	-29.64	-9.94±0.70
Mamonal 2	Sentinel 1	10.32.71 -75.50.55	**-16.25**	**-1.66±0.45**
	Terrasar X	10.32.71 -75.50.55	3.35	-3.27±2.50
Pasacaballos	Sentinel 1	10.29.76 -75.51.25	**-42.04**	**-8.11±0.48**
	Terrasar X	10.29.76 -75.51.25	21.44	2.53±2.83
Puerto Bahía	Sentinel 1	10.28.27 -75.51.93	**-23.94**	**-3.19±0.53**
	Terrasar X	10.28.27 -75.51.93	7.42	1.18±2.63
Boquilla	Sentinel 1	10.45.69 -75.50.72	**-11.79**	**-1.91±0.34**
	TerraSAR X	10.45.69 -75.50.72	-14.49	-6.20±1.07

Supplementary Table S1. Cumulative values (mm) and rates of subsidence (mm/yr) at selected points in the city of Cartagena from InSAR data, Sentinel-1 (2014-2020) and Terrasar-X (2017-2020). Some stations are displayed in figures 1c and 4.
